# Color Constancy in Two-Dimensional and Three-Dimensional Scenes: Effects of Viewing Methods and Surface Texture

**DOI:** 10.1177/2041669517743522

**Published:** 2017-12-06

**Authors:** Takuma Morimoto, Yoko Mizokami, Hirohisa Yaguchi, Steven L. Buck

**Affiliations:** Faculty of Engineering, Chiba University, Chiba, Japan, Department of Experimental Psychology, 6396 University of Oxford, UK; Graduate School of Engineering, Chiba University, Chiba, Japan; Chiba University, Chiba, Japan; Department of Psychology, University of Washington, Seattle, WA, USA

**Keywords:** 3-D perception, adaptation, constancy, color, object recognition

## Abstract

There has been debate about how and why color constancy may be better in three-dimensional (3-D) scenes than in two-dimensional (2-D) scenes. Although some studies have shown better color constancy for 3-D conditions, the role of specific cues remains unclear. In this study, we compared color constancy for a 3-D miniature room (a real scene consisting of actual objects) and 2-D still images of that room presented on a monitor using three viewing methods: binocular viewing, monocular viewing, and head movement. We found that color constancy was better for the 3-D room; however, color constancy for the 2-D image improved when the viewing method caused the scene to be perceived more like a 3-D scene. Separate measurements of the perceptual 3-D effect of each viewing method also supported these results. An additional experiment comparing a miniature room and its image with and without texture suggested that surface texture of scene objects contributes to color constancy.

## Introduction

We tend to perceive the surface color of an object consistently despite changes in scene illumination. This visual property is known as color constancy. It is often explained by relatively lower level mechanisms, for example, von Kries’ (1970) adaptation in the retina. However, some studies suggest an influence of spatial structure on color appearance (Bloj, Kersten, & Hurlbert, 1999; Boyaci, Doerschner, Snyder, & Maloney, 2006; Ikeda, 2004; Yamauchi & Uchikawa, 2005). Other studies have shown the influence of three dimensionality or depth cues on color constancy. For example, Hedrich, Bloj, and Ruppertsberg (2009) showed better color constancy for three-dimensional (3-D) scenes using real objects. Yang and Shevell (2002) used two-dimensional (2-D) images generated by computer graphics and showed that binocular disparity and specular highlights improve color constancy in a 2-D scene. In addition, Xiao, Hurst, Maclntyre, and Brainard (2012) showed that the 3-D shape (e.g., a sphere) of the objects and the presence of specular highlights could be cues to estimate the illuminant under their reduced cue condition. Werner (2006) found that the degree of color constancy is influenced by depth segmentation. Mizokami and Yaguchi (2014) showed that recognizing spatial structure plays an important role in achieving color constancy. Moreover, Mizokami, Ikeda, and Shinoda (2004) and Phuangsuwan, Ikeda, and Katemake (2013) indicated that color constancy could be improved if a photograph is perceived as a 3-D scene by means of a dimension-up viewing box. This tool is a black box into which the observer inserts his or her head and views a 2-D picture monocularly through a rectangular window, with no visible surrounding 3-D structure. This removes binocular-disparity cues from the image, which could otherwise give information that the photograph is a flat surface, and enhances observers’ perception of the picture as a 3-D scene. Their results suggest that color constancy can be improved even if the scene is not an actual 3-D scene. Collectively, these prior studies support the idea that color constancy is better in 3-D scenes than in 2-D scenes.

On the other hand, some studies (Almeida, Fiadeiro, & Nascimento, 2010; Kraft, Maloney, & Brainard, 2002) have shown that there is little influence of scene dimensionality or depth cues on color constancy. In addition, in a relatively recent review paper, Foster (2011) argued that degrees of color constancy are not systematically different between real and simulated scenes. Thus, it is still an open question whether scene dimensionality might affect color constancy.

One could argue that these inconsistent observations might stem from methodological differences. Indeed, these studies employed a wide variety of methods to measure the degree of color constancy: color memory (Hedrich et al., 2009); achromatic setting (Kraft et al., 2002; Mizokami et al., 2004; Yang & Shevell, 2002); two-alternative forced choice task of red–green or blue–yellow (Werner, 2006; Xiao et al., 2012); color judgment choosing one or two hues from red, green, blue, and yellow (Mizokami & Yaguchi, 2014); illuminant color matching (Phuangsuwan et al., 2013); and discrimination between material and illuminant changes (Almeida et al., 2010). Furthermore, what are considered to be *2-D* and *3-D* conditions also differ from one study to another. For example, some studies used real scenes for both 3-D and 2-D conditions (e.g., Almeida et al., 2010; Hedrich et al. 2009), whereas other studies used simulated scenes (e.g., Xiao et al., 2012; Yang & Shevell, 2002). Most importantly, most prior studies on differences in color constancy between 2-D and 3-D scenes did not equate both scenes for size, mean chromaticity, mean luminance, and viewing point, differences that confound the investigation of the effect of the scene dimensionality on color constancy. The first purpose of our study was to carry out a direct comparison of color constancy between 2-D and 3-D scenes, where *2-D* and *3-D* scenes are implemented as “simulated scenes on a monitor” and “real scenes consisting of actual objects,” respectively.

In order to do so, we need to equate various factors other than dimensionality that might differentiate 2-D and 3-D scenes, for example, the chromatic and luminance distributions, the visual size of a scene, spatial resolution, and binocular disparity. Also, there is a distortion in a 2-D scene whereby objects look different from those in a 3-D scene in terms of geometry, such as shape, size, or apparent depth (Smith, 1958a, 1958b). Epstein and Rogers (1995) argued that our visual system has a compensation system for this distortion, and the distortion becomes minimal if the position of viewing a picture and taking a picture is the same. This explanation suggests that comparable viewing points would be also an important factor for a direct comparison. Based on these observations, we created a miniature room with actual 3-D objects and displayed its same size image on the monitor for the direct comparison of the degree of color constancy in each scene.

In addition to physically setting up a 3-D scene, another way to test the effects of scene dimensionality would be the manipulation of the degree to which the scene is perceived as 3-D by varying the viewing conditions. Therefore, the second purpose of this study was to investigate the effect of three viewing methods designed to make a 2-D scene appear more like a 3-D scene. In the first method, we used monocular viewing as with the dimension-up box employed by Mizokami et al. (2004). This viewing method removes binocular-disparity cues which would otherwise tell us that the scene is a flat, 2-D image and enhances the perception that we are viewing a 3-D scene. In addition to these static factors, dynamic cues, such as motion parallax, can contribute to 3-D perception of a scene. Thus, for the second viewing method, we adopted a well-known technique used by Rogers and Graham (1979) in which the image on the monitor changes in response to the head movement of the observer to simulate motion parallax. Because motion parallax contributes to 3-D perception, we expected that it could also help to make a 2-D scene look more like a 3-D scene. Finally, for the third viewing method, we employed the binocular-viewing method as a control condition.

In order to assess the perceptual effect of each viewing method, we separately measured the degree of perceptual three-dimensionality effect (how much depth the scene appears to have) for each viewing condition using the method of magnitude estimation in which observers rated the degree of the 3-D effect by assigning numerical values to the 2-D and 3-D scenes with each viewing method.

The third aim of this study is to investigate how color constancy can be influenced by properties of objects within a scene. Some recent studies have shown that specular highlight, texture, or familiarity of objects can change color appearance (Granzier, Vergne, & Gegenfurtner, 2014; Hansen, Olkkonen, Walter, & Gegenfurtner, 2006; Ling & Hurlbert, 2004; Olkkonen, Hansen, & Gegenfurtner, 2008; Vurro, Ling, & Hurlbert, 2013; Xiao & Brainard, 2008; Xiao et al., 2012). Specifically, we investigated whether the surface texture of objects in a scene might also influence color constancy by comparing scenes in which objects had either homogeneous or naturally textured surfaces.

## Methods

### Apparatus and Stimuli

**Figure 1.**
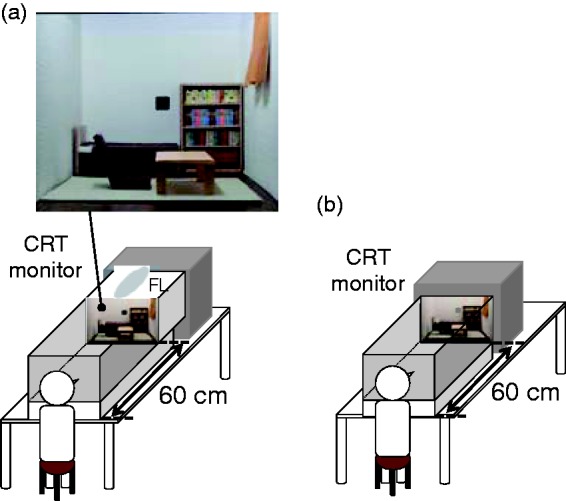
Experimental environment. (a) In the 3-D condition, observers viewed the miniature room illuminated by a fluorescent lamp (FL). Test colors were presented on a CRT monitor behind the miniature room through an aperture on the back wall. (b) In the 2-D condition, the image of the miniature room was presented on the CRT monitor.

Stimulus conditions were divided into 3-D and 2-D conditions. For the 3-D condition, we prepared a miniature room that imitated a natural living room, as shown in Figure 1(a). All objects, floors, and walls were created with matte surfaces to avoid highlights. Its size was 37 cm W × 25 cm H × 50 cm D (34.3° W × 23.5° H). An observer sat on a chair with his or her head fixed by a chin rest situated 60 cm from the front of the miniature room, which was illuminated by a ceiling fluorescent lamp through a translucent acrylic plate placed on the ceiling of the miniature room to diffuse the illuminant. The ceiling was not visible to the observer. The miniature room had a 1.5 × 1.5° square aperture on the center of the back wall through which the test patch was presented via a 21-in. CRT monitor (View Sonic, G225f, resolution 1024 × 768 pixels, calibrated by Color-CAL (Cambridge Research System)) located behind the miniature room.

To create the 2-D condition, we first measured the chromaticity and luminance of the actual miniature room with a 2-D color analyzer (CA-2000, KONICA MINOLTA) having a 980 × 980 pixels resolution set at the position of the observer. We then created a still image on the monitor by converting the measured chromaticity and luminance data into monitor RGB and resized it to 887 × 600 pixels (using a nearest neighbor interpolation method) to make its visual angle the same as the 3-D scene. Note that the same CRT monitor was used to present the test image in the 2-D condition and the test patch in the 3-D condition.

Accuracy of color reproduction is important for direct comparison of the effects of 2-D and 3-D scenes because a difference of chromaticity distribution may cause differences in illuminant perception. If we had used a regular digital camera to take a picture of the 3-D miniature room, we would need to convert the camera RGB space to the monitor RGB space. The accuracy of color reproduction would depend on the accuracy of the conversion. In contrast, the 2-D color analyzer could directly measure the chromaticity and luminance to allow for a more accurate conversion to the monitor RGB space. Thus, although the 2-D color analyzer could sample only 980 × 980 pixels, we employed it instead of a higher resolution digital camera in order to reproduce the chromaticity and luminance of the 3-D miniature room more accurately.

The 34.3° × 23.5° 2-D test image was presented on the CRT monitor via a visual stimulus generator (Cambridge Research System, ViSaGe) with the remaining area on the CRT monitor masked by a black board to be invisible to observers. As shown in Figure 1(b), for 2-D conditions, the front of the CRT monitor was set at the same distance (60 cm) from the observer as the front of the 3-D miniature room to equate their viewpoints. The 1.5 × 1.5° test patch was presented at the center of the image. The experimental environment was dark and surrounded by black walls, so nothing except for the test scene was visible to the observer.

**Figure 2.**
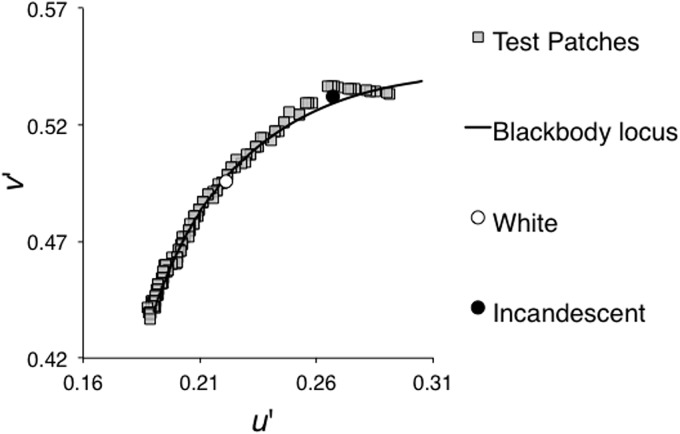
Gray squares indicate the measured chromaticity of the test patch, which was adjustable between 2,000 K and 10,000 K with 50 K steps. White and black circles indicate white points of the white and incandescent illuminants, respectively. The black curve shows the blackbody locus.

A test patch was presented at a randomly selected color temperature between 2,000 K and 10,000 K. The luminance of the test patch was constant at 12.6 cd/m^2^ (corresponding to the luminance of Value 6 color chip in the Munsell color system under a neutral white fluorescent light that is detailed later) and its chromaticity was adjustable along the blackbody locus from 2,000 K to 10,000 K in 50 K steps, as shown by gray squares in Figure 2. The linear scaling in correlated color temperature does not guarantee perceptually equal steps, but we confirmed that these steps were small enough for satisfactory matching, and observers had no trouble finding the achromatic points. Thus, the choice of the scale did not affect the trend of our results.

**Figure 3.**
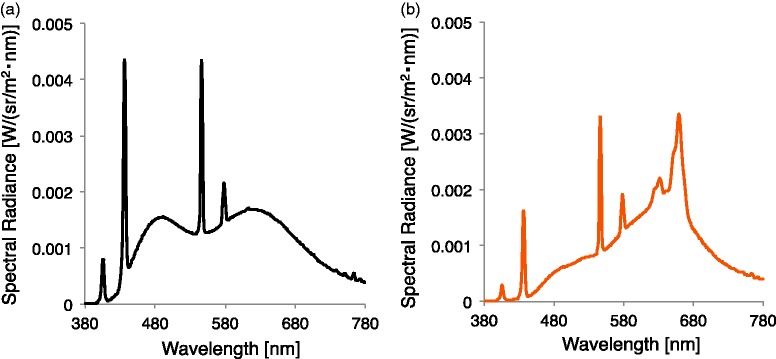
Spectral energy distribution for two test illuminants. (a) Neutral white fluorescent light and (b) incandescent color type fluorescent light.

We used two colors of test illuminants, a neutral white fluorescent light (TOSHIBA, FL20S-N-EDL, 4640 K correlated color temperature, Ra99) and an incandescent color type fluorescent light (Panasonic, FL20S-L-EDL, 2690 K, Ra95). Figure 3 shows spectral energy distributions of both illuminants. The horizontal illuminance was roughly 400 lx at the height of the test patch under both illuminants. In the miniature room, we measured the color of each illuminant by measuring the chromaticity of a white calibration plate (KONICA MINOLTA, CS-A20) set on the floor of the miniature room at a 45° angle to the horizontal plane. These illumination colors determine the white point in the 3-D scene. To determine the white point in the 2-D scene, we created an image of the miniature room, including the white calibration plate and measured the chromaticity of the calibration plate in the image presented on the CRT monitor. We defined these chromaticities as the white points for the 2-D scene. Table 1 summarizes the white point of each illuminant in the 2-D condition and the 3-D condition.

**Table 1.** Mean Chromaticity and Luminance Statistics and White Point of Each Illuminant of 3-D and 2-D Scenes.

Illuminant	White		Incandescent	
Scene condition	3-D	2-D	3-D	2-D
Mean chromaticity				
*u*′	0.227	0.229	0.285	0.283
*v*′	0.505	0.502	0.531	0.531
Illuminant white point				
*u*′	0.221	0.226	0.267	0.276
*v*′	0.496	0.492	0.532	0.534
Lowest luminance (cd/m^2^)	1.04	0.80	1.05	0.80
Highest luminance (cd/m^2^)	97.3	83.3	88.9	67.2
Mean luminance (cd/m^2^)	31.4	28.4	31.8	28.8

*Note.* Those in 3-D and 2-D scenes were roughly the same. 3-D = three dimensional; 2-D = two dimensional.

**Figure 4.**
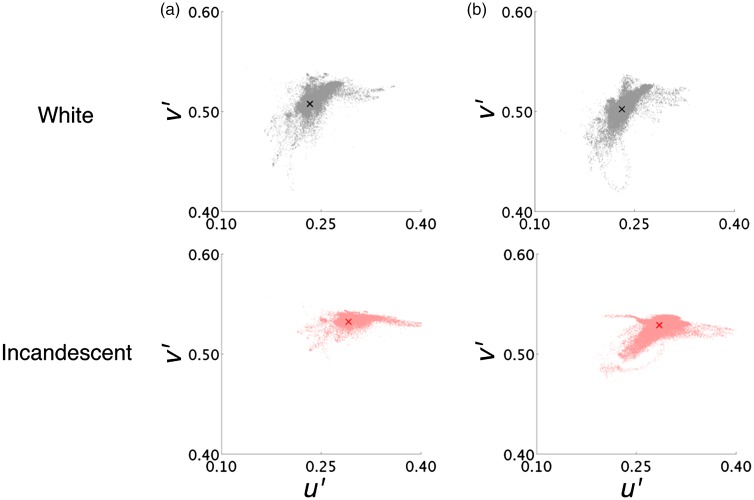
Chromatic distributions for (a) 3-D scene and (b) 2-D scene. Gray and red dots indicate chromaticity sampled by CA-2000 under white and incandescent illuminants, respectively. Cross indicates mean chromaticity.

Table 1 also shows that although the highest luminance was lower in the 2-D condition because some bright colors were out of the monitor gamut, mean chromaticity, mean luminance, and white point were comparable in both scenes. Matching these statistics would not guarantee the perfect comparability for the 2-D and 3-D scenes, but it is worth noting that both scenes had almost the same visual angle, mean chromaticity, mean luminance, and viewing position in order to equate lower level visual processing of the scenes as much as possible. Figure 4 also shows the chromatic distributions for (a) 3-D and (b) 2-D conditions under both test illuminants.

**Figure 5.**
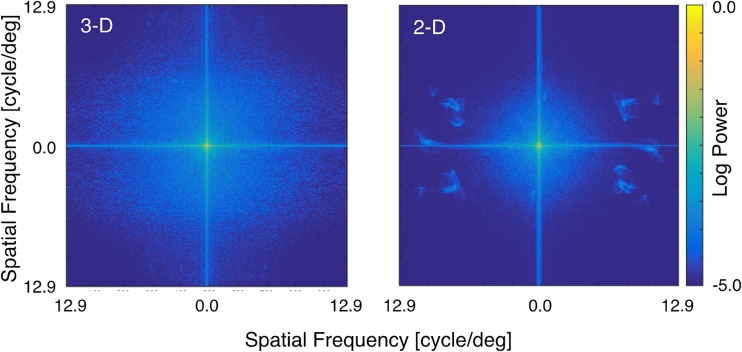
Spatial frequency characteristics in 3-D scene (left panel) and 2-D scene (right panel). 3-D = three dimensional; 2-D = two dimensional.

However, the limited spatial resolution of the 2-D image (887 × 600 pixels) meant that we could not veridically replicate all details of surface texture found in the 3-D scene. As suggested by the results of Vurro et al. (2013) and Xiao et al. (2012), surface texture could be an important factor in color constancy. Thus, we were concerned that the loss of texture on the surfaces of the objects in the 2-D condition might confound effects of scene dimensionality on color constancy. Figure 5 shows the spatial frequency characteristics of (a) the 3-D scene and (b) the 2-D scene under the white illuminant, calculated by MATLAB functions based on the luminance measurements made with the 2-D color analyzer. Horizontal and vertical axes show the power density of horizontal and vertical spatial frequencies, respectively. Thus, the center region indicates low spatial frequencies, and the greater is the distance from the center, the higher is the spatial frequency. The power at each spatial frequency is expressed as color, and the maximum power was normalized to 1.0 for each 2-D and 3-D scene. If the 3-D miniature room was veridically reproduced on the CRT display, the spatial frequency characteristics in (a) 3-D and (b) 2-D scenes would be identical. However, Figure 5 shows that the power of middle to high spatial frequencies in the 2-D scene is lower than in the 3-D scene.

**Figure 6.**
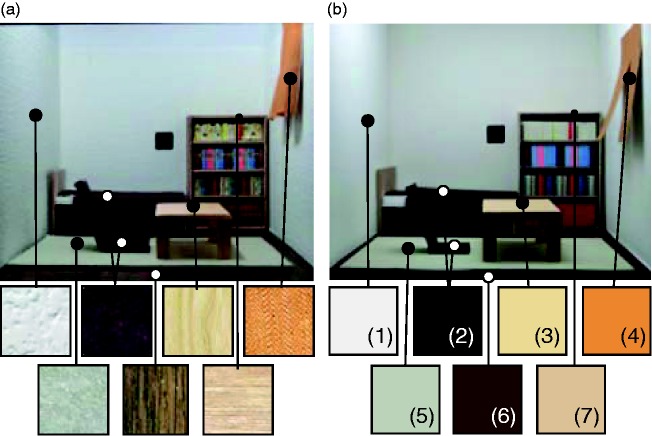
(a) The original room for the comparison and (b) the room employed in additional experiment. Objects, walls and floor had no texture in the room (b).

**Figure 7.**
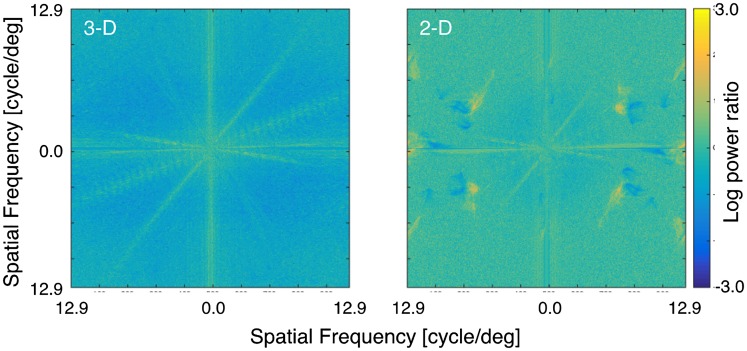
Logarithm of power ratio between texture and no-texture conditions for 3-D scene (left panel) and 2-D scene (right panel). 3-D = three dimensional; 2-D = two dimensional.

This spatial frequency analysis motivated us to further investigate whether loss of texture influences color constancy. This involved constructing a new room in which objects had no texture on their surfaces but equivalent color properties to the originals. We first measured the mean Munsell color notation of each texture in the 3-D room using a spectrophotometer CM-3600d (KONICA MINOLTA). Then, we constructed the new room using customized matte color papers (made by the Japanese Color Research Institute) having those Munsell color notations. Figure 6 shows both the original room and the new room created with homogeneous color and no highlights on its surfaces. Figure 7 allows us to see changes in spatial frequency content caused by removing textures. As shown in the color bar on the right side, color expresses the ratio of power between the texture and no-texture conditions at each spatial frequency. Thus, if power is the same for texture and no-texture conditions, the logarithm of the power ratio is zero. Positive values (yellow direction) mean that the power in the no-texture condition is higher than in texture condition and negative values (blue direction) mean the other way around. For 3-D scenes, the power ratio from middle to high frequencies is negative (bluish), which means that the no-texture condition has less power than the texture condition in the high frequency region. In contrast, for 2-D scenes, the power ratios seem close to zero (greenish) for most spatial frequencies, demonstrating that the spatial-frequency composition of texture and no-texture conditions is relatively similar to each other in the 2-D condition.

Table 2 shows the mean Munsell color notations of textured surfaces and corresponding color papers measured by the spectrophotometer CM-3600d. Although their hues are slightly different, they are close to each other. Mean chromaticity and luminance in both scenes are shown in Table 3. They are comparable between 3-D and 2-D scenes and also between texture (Table 1) and no-texture conditions. For this additional experiment, the achromatic point was measured using the same method as in the main experiment. Observers and procedure were the same as in the main experiment, but we employed only the binocular-viewing condition.

**Table 2.** Munsell Notation (Hue Value/Chroma) of Each Object in Texture Room and No-Texture Room.

No.	Textured surfaces	Homogeneous surfaces
1	4.8Y 9.4/0.6	4.2Y 9.3/0.7
2	4.3YR 3.2/2.3	3.9YR 3.2/2.3
3	6.7YR 5.8/6.2	6.6YR 5.8/6.0
4	0.1YR 2.9/1.5	9.9R 2.9/1.5
5	9.5YR 7.5/4.1	9.3YR 7.4/4.0
6	8.0YR 6.7/3.2	7.8YR 6.6/3.1
7	5.1Y 7.3/1.9	4.7Y 7.2/1.8

**Table 3.** Mean Chromaticity and Luminance of No-Texture Room Used in Additional Experiment.

Illuminant	White		Incandescent	
Scene condition	3-D	2-D	3-D	2-D
Mean chromaticity				
*u*′	0.228	0.236	0.285	0.291
*v*′	0.505	0.504	0.531	0.531
Mean luminance (cd/m^2^)				
*L*′	34.7	33.5	34.3	32.5

*Note*. Those of 3-D and 2-D were roughly the same under each illuminant. 3-D = three dimensional; 2-D = two dimensional.

### Viewing Conditions

To examine whether color constancy is improved when the scene is perceived as more like a 3-D scene, we employed three viewing conditions detailed in the following. Note that all viewing conditions were used for both the 3-D and the 2-D conditions to make a comparison.

#### Binocular viewing

Observers viewed the test scene with both eyes. In the 3-D scene condition, it was expected that the scene would look like a space with depth because of differences of binocular disparity for the different parts of the room. In contrast, the 2-D scene was expected to appear as a 2-D scene under binocular-viewing conditions because no differences of binocular disparity were produced.

#### Monocular viewing

This condition was intended to make the 2-D scene more like the 3-D space by eliminating the possibility and expectation of binocular disparity, which would otherwise provide cues that the image on the monitor was flat. Observers saw the test scene with their left eye patched, so it was expected that the 2-D scene would look more 3-D than in the binocular-viewing condition.

#### Head movement

For this condition, an observer’s chin rested on a horizontally movable chin rest whose range of motion was 13 cm. We asked observers to view the stimulus binocularly. In the 2-D condition, when observers moved their head, the image on the monitor was updated in response to the head movement to simulate motion parallax based on the estimated position of the head of the observer. This condition was intended to add dynamic depth cues to the 2-D scene to make it look like a 3-D scene. This motion parallax was simulated by 32 possible images from one end of the head movement to another. Although it was recognizable that it was a simulated scene rather than a real room, all observers agreed that the simulation induced a perceptual 3-D effect.

### Observers

Three men (D. T., H. T., and T. M.) and two women (M. K. and T. K.) observers, who were 20 to 29 years of age and had normal color vision assessed by Ishihara plates (Ishihara, 2007), participated. T. M. was an author. Observers except for T. M. were unaware of the way illuminants changed the surface colors, and T. K. was also naïve about the purpose.

### Procedures

We employed an achromatic setting technique to determine the neutral perception point in each scene. After 2 minutes of dark adaptation followed by 2 minutes of adaptation to the scene illuminant, the observer adjusted the chromaticity of the test patch with a keyboard until it appeared achromatic, without time limitation. There was no intertrial interval between settings, and the color temperature of the test patch was reset randomly between 2,000 K and 10,000 K before each setting. We asked the observers to look around the scene while adjusting the test patch in order to minimize local retinal adaptation to the scene and test patch. For head-movement condition, to standardize head motion, the observer was instructed to move his or her head smoothly over the full distance of 13 cm once each second, as cued by a 1 Hz tone, while adjusting the chromaticity of the test patch.

Each session had 12 blocks consisting of a combination of two illuminant conditions, three viewing conditions, and two scene dimensionalities (3-D or 2-D). Each block consisted of five successive achromatic settings without an intertrial interval. All observers performed five sessions altogether. The order of conditions was fully randomized.

After completion of all sessions for the main experiment (effects of viewing methods), we additionally measured the perceptual 3-D effect for each viewing condition in 2-D and 3-D scenes based on a magnitude-estimation method. Observers were asked to assess how 3-D each scene looked for each viewing condition, with the 3-D room viewed with binocular viewing defined as 10. This evaluation was repeated three times.

Finally, the observers conducted the additional experiments using the room with no textures on objects’ surface with the same procedure as in main experiment. There were four conditions: the combination of two illuminant conditions (white and incandescent) and two scene dimensionalities (3-D or 2-D). Only binocular viewing was employed. The order was randomized.

## Results

### Effects of Scene Dimensionality and Viewing Methods

**Figure 8.**
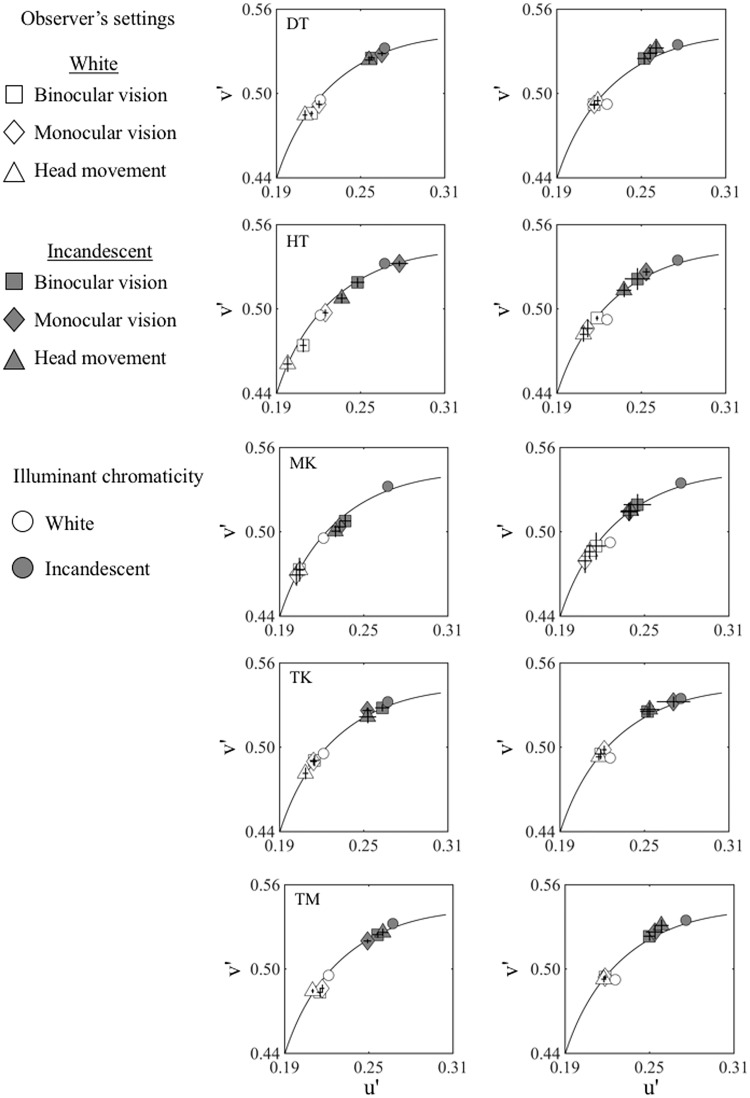
Mean achromatic settings of all observers in CIE1976 *u′v*′ chromaticity diagram. Squares, triangles, and diamonds correspond to binocular, monocular, and head movement, respectively. Small open and black-filled circles show the white points under white and incandescent illuminant, respectively. Open symbols show settings under white illuminant and gray-filled symbols show settings under incandescent illuminant. The black solid line shows the black body locus. The error bars indicate ± *SD* across sessions.

Figure 8 shows the mean achromatic settings of the test patch under all viewing and illuminant conditions for all observers in the CIE1976 *u′v*′ chromaticity diagram. Squares, diamonds, and triangles indicate binocular-viewing, monocular-viewing, and head-movement conditions, respectively. Open symbols indicate the results under the white illuminant and gray-filled symbols indicate the results under the incandescent illuminant. In addition, the white points of the two illuminants are shown as small open (white) and black-filled (incandescent) circles. Each point represents the mean of 25 settings. The black solid line in each panel indicates the black body locus. The error bars indicate ±*SD* across sessions.

The method for quantification of color constancy is still controversial (e.g., see Foster, 2011). However, the degree of color constancy is generally analyzed by the ratio between the perceptual and physical shifts of white points caused by illuminant change. In this study, to quantitatively compare the degree of color constancy between 2-D and 3-D scenes and among viewing conditions, we defined a color constancy index (CCI), which ranged from 0 (no constancy) to 1 (perfect constancy: same amount of shift as illuminant color), as shown in Equation (1). In Equation (1), *a* is the Euclidean distance between the achromatic settings under the two illuminants, and *b* is the Euclidean distance between the colorimetric white points of the two illuminants. This calculation was performed on the CIE *u′v*′ chromaticity diagram.

(1) CCI=a/b

**Figure 9.**
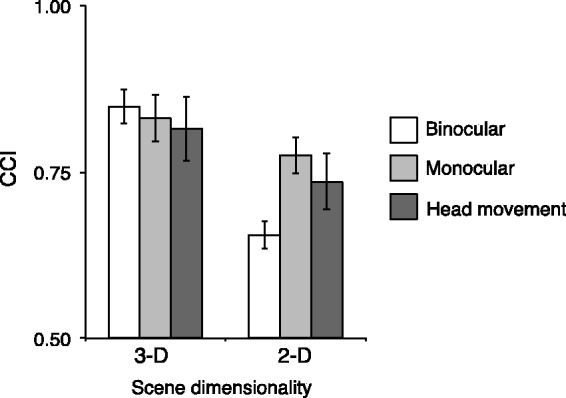
Color constancy indices (CCI) of 3-D and 2-D scene conditions for each viewing method. The higher the value is, the better the color constancy works. Indices are averaged across five observers. Error bars indicate ± 1 SEM. 3-D = three dimensional; 2-D = two dimensional.

From Equation (1), we calculated the CCI for 2-D and 3-D scenes in each of the viewing conditions as shown in Figure 9. Error bars indicate the standard errors across observers.

We performed a two-way repeated-measures analysis of variance, with scene dimensionality (3-D or 2-D) and viewing conditions (binocular, monocular, and head movement) as the within-subject factors. First of all, we found significant main effects of scene dimensionality, *F*(1, 4) = 60.39, *p* < .005, indicating the superior color constancy in the 3-D condition, while the main effects of viewing conditions were not significant, *F*(2, 4) = 1.046, *p* >.1.

Second, there was a significant interaction between the scene dimensionality and the viewing conditions, *F*(2, 8) = 60.39, *p* < .005. The analysis of the simple main effect revealed CCI of 3-D conditions significantly differed from CCI of 2-D conditions for every viewing condition: binocular viewing, monocular viewing, and head movement, *F*(1, 4) = 65.9, *p* < .001, *F*(1, 4) = 5.546, *p* < .05, and *F*(1, 4) = 11.0, *p* < .01, respectively. Importantly, viewing conditions had a simple main effect on the CCI for 2-D conditions, *F*(2, 8) = 4.869, *p* < .05, indicating the viewing method affected color constancy in the 2-D scene but not for 3-D condition, *F*(2, 8) = 0.373, *p* >.1.

Finally, multiple comparisons using Ryan’s method revealed that, for the 2-D condition, the CCI was significantly higher for monocular viewing than for binocular viewing, *t*(4) = 3.061, adjusted *p* < .05. However, there was no significant difference between binocular-viewing and head-movement conditions or between monocular-viewing and head-movement conditions, *t*(4) = 2.056, adjusted *p* >.05; *t*(4) = 1.005, adjusted *p* >.05.

We found that the CCI was greater in 3-D scenes than in 2-D scenes for all viewing conditions. This suggests that scene dimensionality surely affects color constancy at least in our instantiation of 2-D and 3-D scenes.

For the 2-D conditions, we compared the relative improvement provided by monocular viewing and head movement over binocular viewing to examine the contribution of each to color constancy. The improvement in CCI with monocular viewing varied between 11% and 43% for individual observers and 19% for the mean across all observers. On the other hand, the improvement with head movement varied from 5% to 29% for an individual observer and 12% for the mean across all observers.

### Perceptual 3-D Estimations

**Figure 10.**
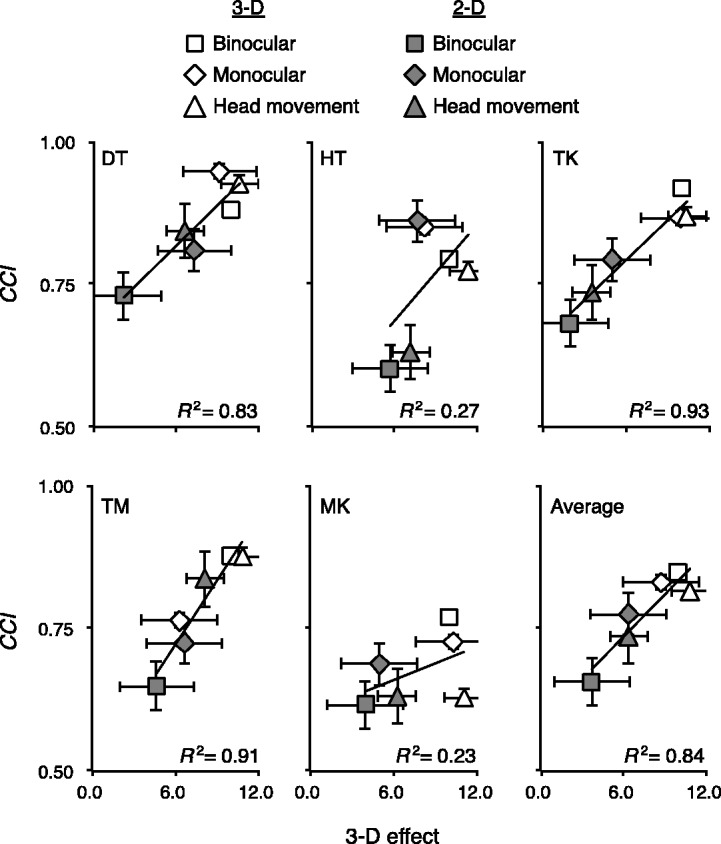
The relationship between CCI and perceptual 3-D effect. The 3-D effect in the 3-D scene with binocular viewing was defined as 10.0. Error bars indicate ± 1 SE. 3-D = three dimensional; CCI = color constancy indices.

One potential concern is that observers might not have actually perceived the 2-D scene as appearing more like 3-D under the monocular-viewing or head-movement conditions. Thus, we measured the perceptual 3-D effects using a magnitude estimation method. Figure 10 allows us to see the correlation between 3-D effect, as assessed by magnitude estimates, and CCI. The horizontal and vertical error bars indicate the standard error of 3-D effect and CCI, respectively. The positive slope of the linear regression lines for all observers shows that perceived three-dimensionality and color constancy preservation are positively correlated. The correlation for the averaged data was statistically significant, *t*(4) = 4.63, *p* < .01.

For 2-D scenes (gray-filled symbols), monocular-viewing and head-movement conditions had greater 3-D effect than the binocular-viewing condition for all observers. Thus, color constancy can work well when the scene is perceived as more 3-D, even when it is actually a 2-D image. For 3-D scenes (open symbols), the 3-D effect for all viewing conditions was higher than in 2-D scenes for the mean and all observers except for T. M. Interestingly, even with monocular viewing, 3-D scenes still had high 3-D effect for most observers.

### Influence of Surface Texture of Scene Objects

**Figure 11.**
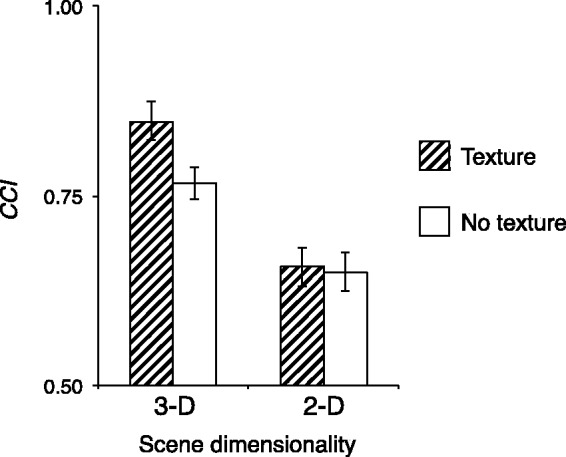
Color constancy indices (CCI) of 3-D and 2-D scene conditions for texture and no-texture conditions. The higher the value is, the better the color constancy. Indices are averaged across five observers. Error bars indicate ± 1 SEM. 3-D = three dimensional; 2-D = two dimensional.

Figure 11 shows the average CCI calculated from Equation (1) for texture (original room from main experiment) and no-texture conditions (new room). There was significantly higher CCI for the texture condition than the no-texture condition for 3-D scene, *t*(4) = 7.06, *p* < .01, whereas there was no significant difference between texture and no-texture conditions for 2-D scene, *t*(4) = 0.184, *p* >.1. This result might imply that the resolution of the 2-D image on the display was not high enough to reproduce texture. Thus, insufficient reproduction of texture appears to be one of the reasons why color constancy in the 2-D scene was not as good as in the 3-D scene, even with monocular viewing.

## Discussion

We found that color constancy for the 2-D scene improved for monocular viewing even though the scene was not an actual 3-D scene. More importantly, we showed a positive correlation between “perceptual” 3-D effects and color constancy. Although the generality of our conclusion is yet to be determined beyond the types of scenes tested here, this study suggests that color constancy is influenced by perceived scene dimensionality.

Color constancy is often explained by low-level (retinal) mechanisms, but our results pose difficulties for such explanations because our 3-D and 2-D scenes were highly comparable in terms of visual size, chromatic and luminance statistics, and viewing point. Therefore, the effect of retinal adaptation, which can have a strong impact on color constancy (Smithson & Zaidi, 2004), would be ruled out to account for the different color constancy between 2-D and 3-D scenes observed in this study. Similarly, statistical-based color constancy models (Brainard & Freeman, 1997; Foster & Nascimento, 1994; Golz & MacLeod, 2002; Land, 1977; Maloney & Wandell, 1986; Uchikawa, Fukuda, Kitazawa, & MacLeod, 2012) would not account for our results because the chromaticity and luminance were equivalent in our 2-D and 3-D scenes, and those models give the same predictions for both scenes. Consequently, our results suggest that color constancy is determined not solely by lower level retinal and early postreceptoral processing but also by higher level cognitive processing such as 3-D perception in scenes.

We expected that head movement could also contribute to color constancy for the 2-D scene, but its CCI was not significantly higher than with binocular viewing. One potential concern about our simulation of the motion parallax was the lack of reality. Although all observers reported higher perceptual 3-D effect for the head-movement condition than for the binocular-viewing condition, it was noticeable that the motion parallax was just a simulation. This lack of reality could be the reason why color constancy did not significantly improve. Alternatively, this could be simply due to greater variability across observers compared to binocular-viewing or monocular-viewing conditions.

One might expect that the loss of binocular-disparity information in the monocular condition would degrade color constancy for the 3-D scene but that was not observed. Instead, almost all of the observers reported that the 3-D scene was perceived as a 3-D scene even with monocular viewing. Possibly, even without binocular disparity, there are enough 3-D cues such as pictorial cues and accommodation.

Unlike our results, Foster (2011) recently summarized research on color constancy and concluded that there was little difference in the effect on color constancy of real versus simulated scenes, stimulus complexity, geometric versus natural scenes, or 2-D versus 3-D scenes. Nascimento, Almeida, Fiadeiro, and Foster (2005) also noted that scene complexity does not affect color constancy. More importantly, Almeida et al. (2010) showed that scene dimensionality did not significantly influence color constancy. As mentioned in the Introduction section, this discrepancy could arise because of differences between our implementation of 3-D and 2-D scenes and theirs. They used a 3-D scene consisting of 3-D actual objects and a 2-D scene consisting of flat actual objects (see Figure 2 in Almeida et al., 2010). Thus, both their 2-D and 3-D scenes were 3-D scenes by our definition. This study has made a first attempt to equate various factors between different dimensional scenes and made it clearer that scene dimensionality contributes to color constancy.

It is also plausible that this difference across studies could be due to differences of methodology. Our observers made achromatic settings of a target within the scene, whereas their observers were asked to distinguish between “illuminant changes” and “material changes.” The color constancy revealed by our method and theirs corresponds to “phenomenal” and “projective” color constancy, respectively (Reeves, Amano, & Foster, 2008). It was indeed shown that the degree of color constancy tends to be higher for projective color constancy. Therefore, the inconsistent results may stem from different measures of color constancy.

The number of observers employed in this study is relatively low, which might make it difficult to generalize our findings. However, we would emphasize that our major focus in this study was a comparison of color constancy performance across viewing conditions, and performance change across conditions was consistent across observers. Moreover, we analyzed the datasets excluding the observer T. M. (an author), but the only difference was that we did not find the significant difference of constancy index between 3-D and 2-D conditions for monocular-viewing condition. Thus, the overall trends did not change. Nevertheless, we acknowledge that further investigation is desirable.

The present finding also can be a useful clue to understand “#the dress” phenomena in which people perceived the appearance of the dress image differently. It has been widely acknowledged that individual differences are likely to come from a difference in perceived illuminant (Brainard & Hurlbert, 2015). One of the important features in “#the dress” phenomenon was that it spread as a still 2-D image, while people generally agree on the appearance when they see a real dress. Thus, from the perspective of the present findings, the fact that the picture of “the dress” was a 2-D image may have contributed to lack of information for illuminant estimation and the consequent variability of appearance. In other words, in the process of being converted to a still image by a camera, the dress might lose some important cues to support consistent illuminant estimation across individuals, and that could consequently cause the ambiguity of appearance. Interestingly, Hesslinger and Carbon (2016) reported that the ambiguity decreased when the image was spatially scrambled, suggesting that spatial structure plays a significant role. To test this possibility, it would be interesting to compare the individual differences of color constancy between 2-D and 3-D scenes as a future extension of this study. In any case, our findings highlight the importance of recognition of scene structure for resolving illuminant ambiguity and separating illuminant and surface properties.

## Conclusions

We examined the effect of scene dimensionality on color constancy by measuring color constancy for a 3-D miniature room and carefully matched 2-D images presented on a monitor using three viewing methods: binocular viewing, monocular viewing, and head movement. Our results suggest that both real and perceived scene dimensionality contribute to color constancy. In an additional experiment, color constancy in a no-textured scene was worse than in a textured scene under the 3-D condition in a real miniature room, implying that color constancy is also influenced by the properties of the scene objects, such as texture.
